# Oxygen Isotope Fractionation between Carbonate Minerals and Carbonic Acid Systems and Constraints for Environmental Science and Geological Processes

**DOI:** 10.3390/molecules29030698

**Published:** 2024-02-02

**Authors:** Jixi Zhang

**Affiliations:** 1 School of Geography and Environmental Science, Guizhou Normal University/State Engineering Technology Institute for Karst Desertification, Guiyang 550001, China; jixizhang@gznu.edu.cn; 2 School of Karst Science, Guizhou Normal University/State Engineering Technology Institute for Karst Desertification, Guiyang 550001, China

**Keywords:** oxygen isotope fractionation, carbonate mineral, H_2_CO_3_ solution, CO_3_^2−^ solution, Ca(HCO_3_)_2_ solution

## Abstract

The equilibrium oxygen isotope fractionation factor is widely used in geological thermometry. However, under most natural conditions, the oxygen isotope exchange is rare to reach equilibrium. Especially for the complex water–rock interaction process, the contribution of the H_2_CO_3_ solution, CO_3_^2−^ solution, Ca(HCO_3_)_2_ solution, and CaCO_3_ solution to the equilibrium oxygen isotope fractionation factor of this process is poorly understood. In view of this predicament, these key parameters are obtained by ab initio calculations. The results showed that the contributions of different carbonate minerals and different aqueous solutions to the equilibrium oxygen isotope fractionation factor were different. Among all nine carbonate minerals (dolomite, calcite, aragonite, magnesite, siderite, otavite, smithsonite, ankerite, and strontianite), the minerals with the highest and lowest reduced partition function ratios (RPFR) were siderite and strontianite, respectively. At the same time, the RPFR of nitratine, which has the same structure as carbonate, was studied. The RPFRs of the three most widely distributed carbonates in nature (dolomite, calcite, and aragonite) were dolomite > calcite > aragonite. Among the H_2_CO_3_ solution, CO_3_^2−^ solution, Ca(HCO_3_)_2_ solution, and CaCO_3_ solution, the H_2_CO_3_ solution had the strongest ability to enrich ^18^O. In addition, the equilibrium oxygen isotope fractionation factors between aqueous solutions and gas phase species (CO_2_(g), H_2_O(g), and O_2_(g), etc.) were calculated systematically. The results showed that the oxygen isotope fractionation factors between solutions and gas phases were often inconsistent with the temperature change direction and that the kinetic effects played a key role. These theoretical parameters obtained in this study will provide key equilibrium oxygen isotope constraints for water-rock interaction processes.

## 1. Introduction

Oxygen is one of the most widely distributed elements in the earth’s crust and mantle. There are three stable isotopes of oxygen (^16^O, ^17^O, and ^18^O), and the mass difference between these isotopes is relatively large (more than 12.5%). According to the basic theory of mass-dependent fractionation, oxygen isotope fractionation is widespread in various processes in nature because of the large mass difference between different oxygen isotopes. In fact, people have realized that oxygen isotope fractionation has played a vital role in various geological processes, biological processes, and chemical processes for a long time [1,2,3,4,5]. In the field of earth sciences, oxygen isotope fractionation is an important means of studying the oxygen cycle between the atmosphere, oceans, plants, and animals, and can detect the movement and conversion between two different oxygen isotopes [6,7,8]. Oxygen isotope fractionation also plays an important role in the study and detection of climate change and environmental pollution [9,10].

The carbonate system, as well as Its closely related gas phases (CO_2_, O_2_, etc.) and liquid phases (CO_3_^2−^ solution, Ca(HCO_3_)_2_ solution, CaCO_3_ solution, H_2_CO_3_ solution, etc.) are the most important reservoirs of oxygen isotopes, and isotope fractionation between them is very obvious [8,11,12]. However, a proper understanding of these isotopic data requires an understanding of the equilibrium isotope fractionation factors between various carbonate minerals and between carbonate and other phases (oxides and solutions). In addition to the experimental method, the ab initio calculation, based on quantum mechanics, is also a reliable method to obtain the oxygen isotope fractionation factors between these substances (phase states). There are many different methods for the theoretical calculation of isotope fractionation factors between different solid phases. In this study, the volume variable molecular cluster method (VVCM) is adopted to calculate the reduced partition function ratios (RPFR, also called β factor) of oxygen isotopes in solid carbonate minerals, which has been verified by many theoretical studies is reasonable and reliable [13,14,15]. Isotope fractionation between different minerals is calculated based on the difference of harmonic vibration frequencies caused by isotope substitution, and these properties can be completed by theoretical calculation. There have been many successful examples using this method of calculating minerals, such as Gao et al.’s work [16]. The results of these theoretical calculations were compared with existing theoretical experimental and natural sample data involving isotope fractionation factors of carbonate minerals. The results show that the calculated results of this study are in good agreement with some theoretical and experimental data. Although there are some data inconsistencies, the oxygen isotope fractionation factor obtained from theoretical calculations is still a good tool for constraining the temperature dependence of isotope fractionation factors and evaluating different theoretical calculation methods.

There has been a lot of work on the theoretical study of isotopic fractionation factors of carbonate minerals, and it has lasted for a long time [8]. The first theoretical calculation of isotope fractionation data for carbonate minerals was by Urey, whose paper also laid the foundation for theoretical computational geochemistry [17,18]. Since then, more and more works on the oxygen and carbon isotopes of carbonate minerals sprang up [19,20]. According to the Urey model (Born–Oppenheimer approximation), the reduced partition function ratio of a substance is only related to its harmonic vibration frequencies, and the translational and rotational energies contribute very little to it. Schauble et al. also used first-principles molecular dynamics methods to theoretically calculate β values (RPFR) of oxygen and carbon isotopes for carbonate minerals such as calcite, aragonite, dolomite, magnesite, and pyroxene [21,22]. Chacko and Deines used statistical mechanics to calculate the reduced partition function ratios (β value) of oxygen isotopes for a large number of carbonate minerals [8]. The theoretical calculation results of the oxygen isotope fractionation data obtained in this study are also compared with the above theoretical and experimental results.

For the complex chemical reaction between carbonate and water, in addition to the exchange of oxygen isotopes between carbonate minerals and water, other species present in aqueous solutions, such as carbonic acid, bicarbonate aqueous solution, carbonate aqueous solution, etc., will also exchange oxygen isotopes with carbonate minerals. Unfortunately, there is a lack of theoretical research in this area. Meanwhile, oxygen isotope exchange reactions also occur between these aqueous species. Oxygen isotope exchange reactions also occur between aqueous species and gaseous species such as O_2_ and CO_2_. This research also focuses on these interesting microscopic processes of oxygen isotope exchange. On the basis of a large number of previous theoretical and experimental results on oxygen isotope fractionation data between carbonate minerals and water, new and more detailed theoretical data on oxygen isotope exchange processes are provided. This provides a molecular-level explanation for researchers to better understand the mechanism of oxygen isotope fractionation in the complex process of the carbonate-solution-gas system. The results obtained in this work will have important implications for understanding O isotope fractionation in natural systems.

## 2. Results

The RPFR (β) of the oxygen-bearing substances involved in this study were accurately obtained according to Formula (5) of the calculation theory part. The RPFRs of these substances were obtained at temperatures of 0, 25, 50, 100, 150, 200, 300, and 500 °C. The RPFRs of carbonate minerals, aqueous solutions, and gas phases are shown in Table 1. Then, the polynomial relationship between the RPFR (β) and the temperature function ($x=10^{6}/T^{2}$) is given by the following formula:

(1) $$1000\ln RPFR=Ax^{3}+Bx^{2}+Cx+D$$

In Formula (1), *A*, *B*, and *C* are coefficient terms corresponding to *x*^3^, *x*^2^, and *x*, respectively, and *D* is the constant term. “*x*” is the temperature function, i.e., $x=10^{6}/T^{2}$, and *T* is the Kelvin temperature. Table 2 shows the corresponding coefficient terms of the temperature function relations of each carbonate mineral, aqueous solutions, and gas phases. In fitting these curves, the data used are isotopic fractionation data at 0–500 °C. For some of the research systems (such as calcite, dolomite, aragonite, magnesite, CO_2_(g), H_2_O(g), and so on), the previous research data are not fully listed. Those who are interested can refer to the relevant literature, such as Schauble et al.’s researches [21,22]. For gas phase molecules such as CO_2_(g), H_2_O(g), O_2_(g), and CO(g), model construction and frequency calculation methods are relatively simple. Different from the model construction method of gaseous substances, there will be subjective factors of researchers themselves when dealing with condensed phases (liquid and solid phases). From the data given in Table 2 and Table 3, one can obtain isotopic fractionation data (RPFR or β) at any temperature within the temperature range of 0–500 °C. Based on the data obtained in this part, the isotopic fractionation factor α between any two substances can be obtained by Formula (2). To see the change in the isotope fractionation factor more directly, 10001nα was used to describe the oxygen isotope fractionation factor. The mathematical relationship between the isotopic fractionation factor α_A-B_ and the RPFRs (β) of these two substances is:

(2) $$1000ln\alpha_{A-B}=1000ln\frac{\beta_{A}}{\beta_{B}}=1000ln\beta_{A}-1000ln\beta_{B}$$

**Figure 1:**
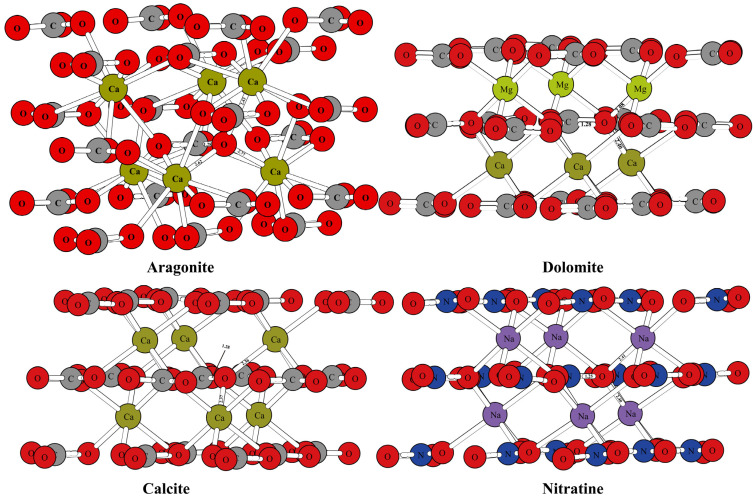
Schematic diagram of mineral structure of aragonite, dolomite, calcite, and nitratine.

**Figure 2:**
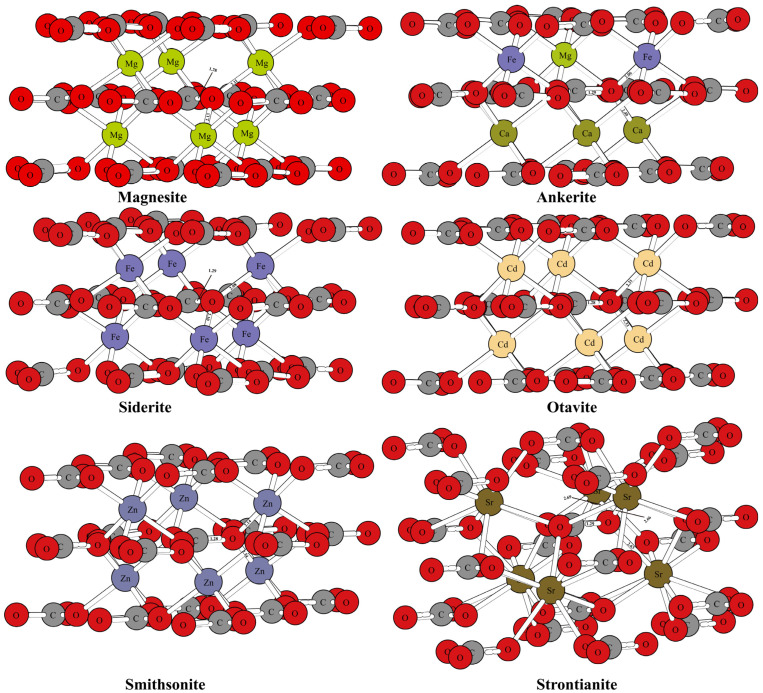
The mineral structures of other carbonate minerals (magnesite, siderite, otavite, smithsonite, strontianite, and ankerite).

**Figure 3:**
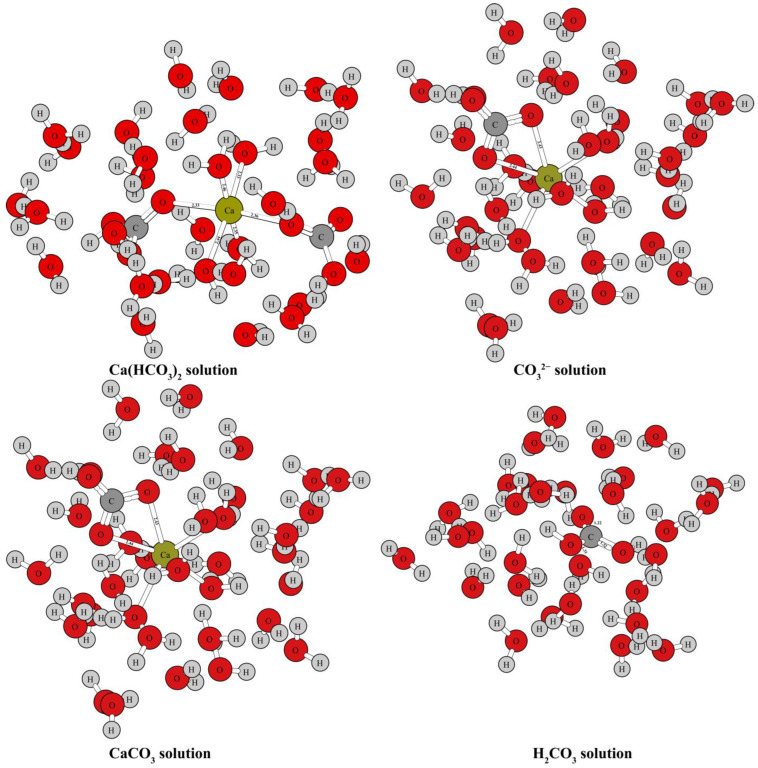
Structure diagram of HCO_3_^−^ (Ca(HCO_3_)_2_) solution, CO_3_^2−^ solution, H_2_CO_3_ solution, and CaCO_3_ solution.

Figure 1 and Figure 2 show the optimized structure of some carbonate minerals. Because there are many kinds of carbonate minerals in this study, only the mineral structures of aragonite, dolomite, calcite, and nitratine are given in Figure 1. The results showed that the differences in the RPFRs of the carbonate minerals were relatively large. Specific data will be explained in detail in the discussion section. Figure 3 gives the structure information of the HCO_3_^−^ (Ca(HCO_3_)_2_) solution, CO_3_^2−^ solution, H_2_CO_3_ solution, and CaCO_3_ solution. The difference of ^18^O enrichment capacity for these aqueous solutions was also very significant.

## 3. Discussion

### 3.1. The Information of Optimized Mineral Structures

The mineral structures of aragonite, dolomite, calcite, and nitratine are shown in Figure 1. The chemical bonds between the oxygen atoms and the surrounding atoms have been marked. The mineral structures of other carbonate minerals (such as magnesite, siderite, otavite, smithsonite, strontianite, and ankerite) are shown in Figure 2. The coordination numbers of the oxygen atoms in different carbonate mineral structures were different. In the crystal structures of calcite, dolomite, and nitratine, oxygen atoms existed in the form of tri-coordination (forming chemical bonds with one carbon or nitrogen atom and two metal atoms). While in aragonite, the oxygen atom formed four chemical bonds with the surrounding atoms (one carbon atom and three metal atoms). On the basis of ensuring the reliability of the calculated data, the atom numbers of the molecular clusters selected were as follows: calcite: 82, aragonite: 90, dolomite: 82, ankerite: 82, magnesite: 82, nitratine: 82, otavite: 82, siderite: 82, smithsonite: 82, and strontianite: 90. The optimized structure information of the mineral structures were compared with the previous literature, as shown in Table 1. As shown in Table 1, the mineral structures treated by VVCM were reasonable, because the change of the chemical bond around the atoms of interest (oxygen atoms) was less than 5% [23,24,25,26]. Therefore, the mineral structures optimized by this method can be used to calculate the harmonic vibration frequencies, because the harmonic vibration frequency is closely related to the chemical bond length.

### 3.2. Optimized Aqueous Solution Structures

Oxygen can be found in many forms in aqueous solutions. There have been many reports on the isotope fractionation effect between carbonate minerals and water [1,8,20,21,22,27,28]. According to the different processes involved in the growth and precipitation of carbonate, an HCO_3_^−^ (Ca(HCO_3_)_2_ solution) solution, CO_3_^2−^ solution, H_2_CO_3_ solution, and CaCO_3_ solution were selected to simulate the oxygen element reservoirs in aqueous solutions. The structure diagram of these four substances is shown in Figure 3. HCO_3_^−^, CO_3_^2−^, H_2_CO_3,_ and CaCO_3_ do not exist in simple forms in aqueous solutions, but as hydrated molecules or hydrated ion groups [29]. This can also be confirmed by the results of structure optimizations and frequency calculations using the “water drop method” (see Figure 3). Oxygen isotope exchange can occur between these aqueous solutions, as well as between substances, solid phases (carbonate minerals), and gas phases (such as CO_2_(g)). Therefore, when discussing oxygen isotope fractionation of water–rock interactions, the processes are very diverse. It is very important to accurately understand the oxygen isotope effect of these processes by correctly obtaining the harmonic frequencies of these substances and then obtaining their RPFRs.

### 3.3. RPFRs of Oxygen-Bearing Substances

**Figure 4:**
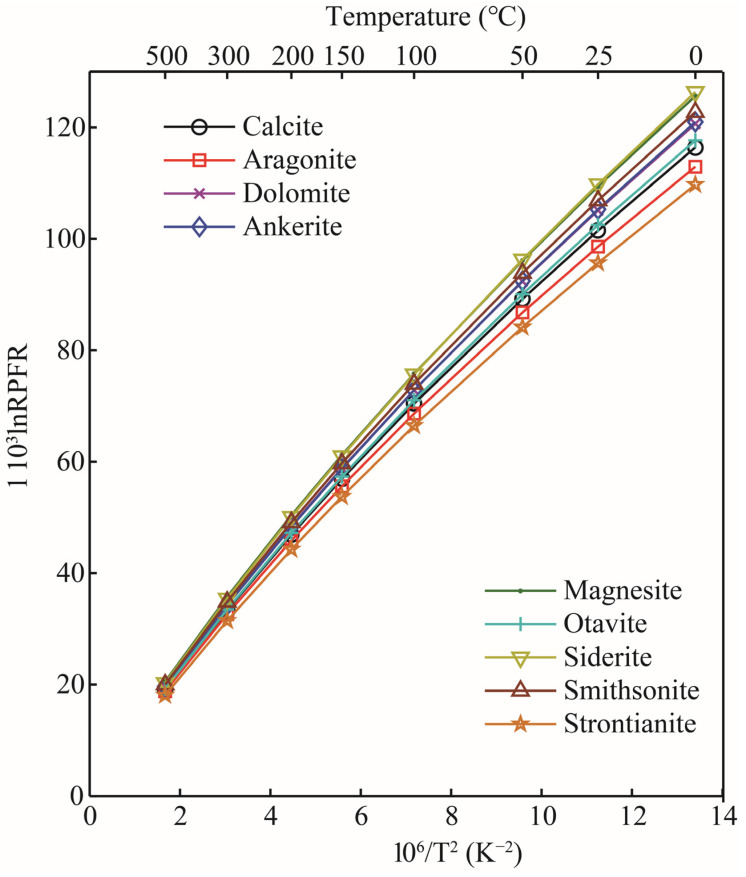
The RPFR_S_ of different carbonate minerals as a function of temperature.

The RPFRs (in the form of $1000\ln RPFR_{18/16}$) for oxygen-bearing solid minerals, aqueous solutions, and gas phase species in the temperature range of 0–500 °C are given in Table 2. According to the data, the $1000\ln RPFR_{18/16}$ of all substances decreased significantly with the increase in temperature. The calculations showed that for oxygen-bearing carbonate minerals, the enrichment sequence of heavy isotope (^18^O) was siderite > magnesite > smithsonite > ankerite ≈ dolomite > otavite > calcite > aragonite > strontianite (see Figure 4). This calculation is in good agreement with the previous studies [21,22]. Taking the 10^3^ln^18/16^β at 0 °C as an example, the values of calcite, aragonite, dolomite, and magnesite calculated in this study were 116.42, 112.95, 120.66, and 125.64 [22], respectively; the corresponding data of Schauble and Young’s results are 114.44, 113.69, 119.53, and 124.82, respectively. The linear fitting formulas between the 10^3^lnRPFR_18/16_ (^18O/16^O) and the temperature function ($x=10^{6}/T^{2}$) of carbonate minerals are shown in Table 3. The results showed that for these four minerals (calcite, aragonite, dolomite, and magnesite), magnesite, calcite, and dolomite were more enriched with ^18^O than aragonite. The 1000lnαs for pairs of calcite-aragonite, dolomite-aragonite, and magnesite-aragonite at 100 °C were 3.46, 7.71, and 12.69, respectively. The reason for such large oxygen isotope fractionation between these minerals is the change in the chemical coordination environments and the types of metal elements in the mineral clusters. From the previous introduction section, we know that the theory of equilibrium isotope fractionation is based on the Born–Oppenheimer approximation and is mass-dependent. At the same time, one of the core points of equilibrium isotope fractionation theory is that the size of equilibrium isotope fractionation is closely related to the chemical bond length. When metal cations in carbonate minerals change, the M–O bond length (M stands for metal element, and the M–O bond length is the length between metal cations and oxygen atoms) is also affected [30,31]. The calculation of carbonate minerals also agrees with this theoretical rule.

**Figure 5:**
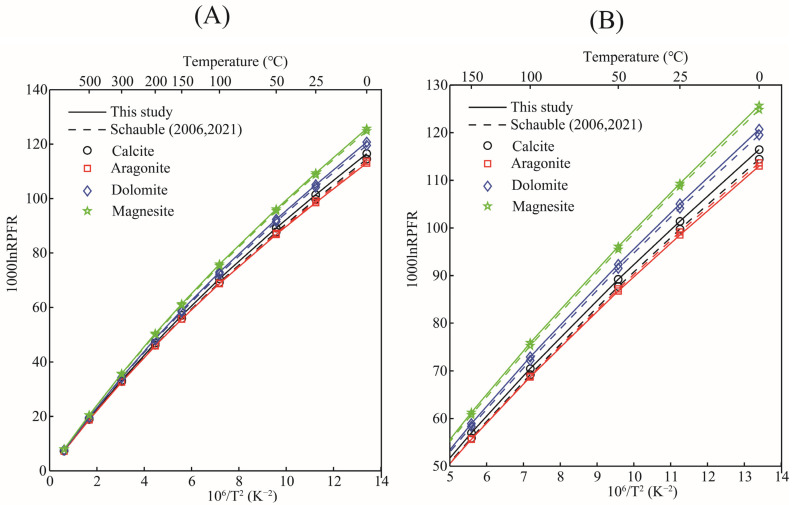
Relation of RPFRs of carbonate minerals calcite, aragonite, dolomite, and magnesite with temperature obtained in this study. The solid line represents the data obtained in this study, and the dashed line represents the data presented in the papers of Schauble et al.’s [21,22]. (**A**,**B**) represent the RPFR change relationship at the temperature range of 0–500 °C and 0–150 °C respectively.

**Figure 6:**
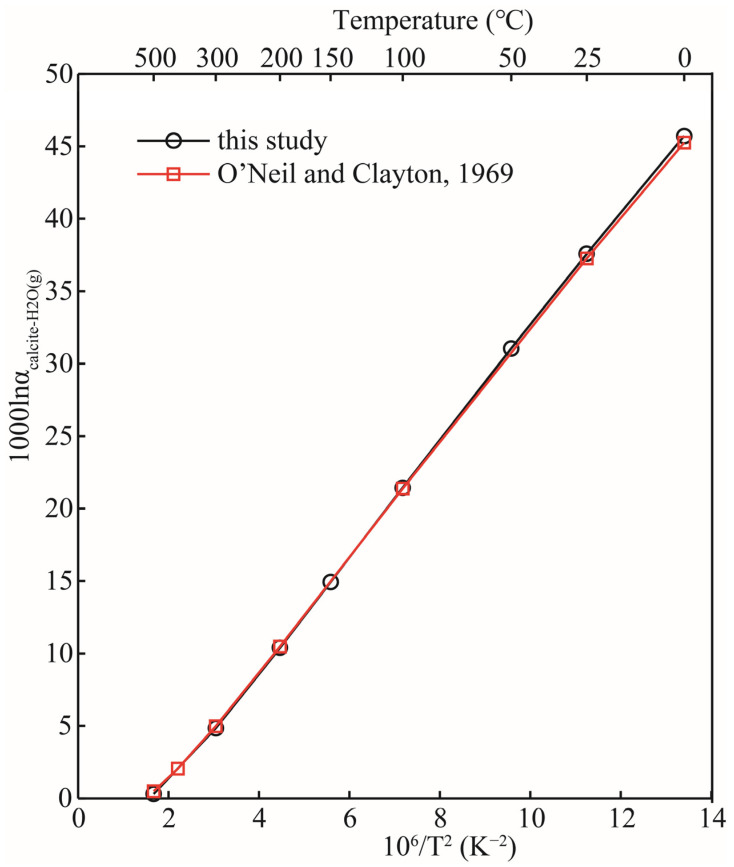
Relation of isotopic fractionation factors between CaCO_3_ (calcite) and H_2_O(g) with temperature. The black and red lines represent the results of this study and previous study [20], respectively.

The 1000lnRPFRs of carbonate minerals obtained in this research, and the data reported in the previous literature, are plotted in Figure 5. As a matter of fact, when discussing equilibrium isotope fractionation, the mere comparison of RPFR is of little significance, but it can still provide certain references such as whether the calculation method is feasible, etc. Figure 5 shows that, with the exception of aragonite, the 10^3^lnRPFRs of calcite, dolomite, and magnesite were systematically higher than those reported by Schauble et al.’s results, due to the different calculation method we adopted [21,22]. Schauble et al. estimated the reduced partition function ratios of carbonate minerals using first-principles lattice dynamics, and the method used in this study was the molecular cluster method (VVCM) based on first-principles calculations. Rather than discussing the differences between RPFRs, it is more meaningful to compare the isotope fractionation factors between them. Taking the isotope fractionation factors between calcite and gaseous water (H_2_O(g)) as an example, the calculated results of this study are in good agreement with previous results (see Figure 6). O’Neil et al.’s research shows that the isotope fractionation factors between CaCO_3_ (calcite) and H_2_O(g) are 45.26, 37.27, 21.38, 10.47, and 4.98 (‰) at 0, 25, 100, 200, and 300 °C respectively [20]. The data obtained in this work were 45.71, 37.58, 21.44, 10.38, and 4.83 respectively under the same temperature conditions. Our results are highly consistent with the data reported in the previous literature [20]. Therefore, there is enough reason to believe that our calculation results are reasonable and reliable.

### 3.4. Oxygen Isotope Fractionation of Carbonate-Carbonate, Carbonate-Aqueous Solutions, Aqueous Solutions-Gas Phases and Aqueous Solutions-Aqueous Solutions

#### 3.4.1. Oxygen Isotope Fractionation between Different Carbonate Minerals

**Figure 7:**
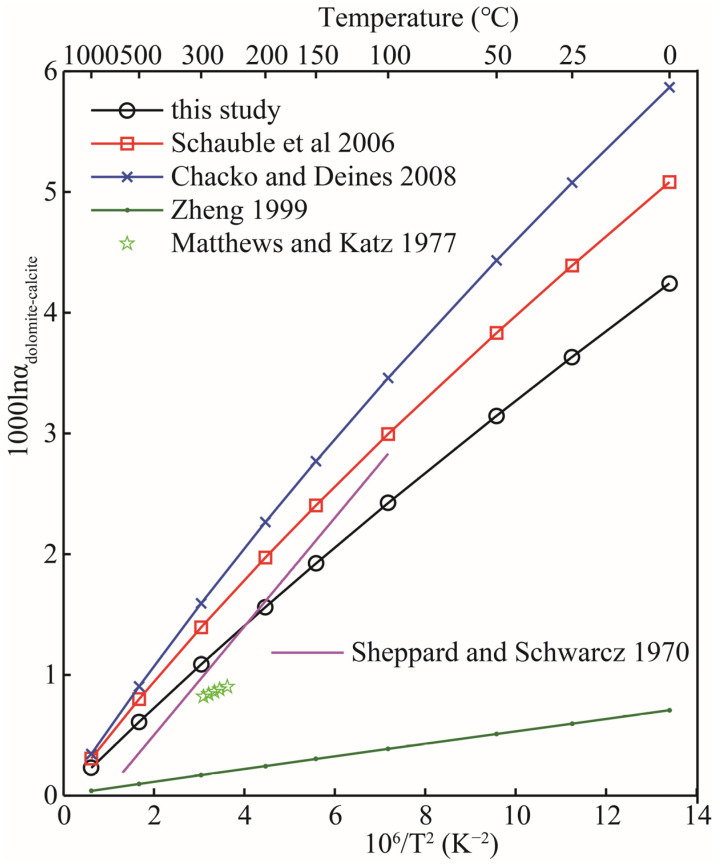
Comparison between the oxygen isotope fractionation factors of dolomite-calcite obtained in this theoretical work and the previous theoretical and experimental results. The black line, red line, blue line, green line, purple line and five-pointed stars represent the results of this study, Schauble et al., Chacko and Deines, Zheng, Sheppard and Schwarcz and Matthews and Katz [4,8,21,32,37], respectively.

The study of oxygen isotopes between carbonate minerals has always been the focus of researchers [32]. Carbonate minerals are the most common minerals in nature. The scientific consensus is that two associated carbonate minerals are a good system for measuring geological temperature [33,34]. The basis of a geothermometer is to accurately obtain the equilibrium isotope fractionation factors between two associated minerals. Unfortunately, until now, there has been no consensus on the magnitude of equilibrium isotope fractionation factors between carbonate minerals [34]. In this part, the dolomite-calcite system is selected as an example to illustrate the oxygen isotope effect between carbonate minerals. The data of oxygen isotope fractionation between other carbonate minerals can also be obtained in sequence according to this method. Figure 7 shows the comparison of our calculation results with previous theoretical and experimental results. Figure 7 makes it clear that the differences between all theoretical and experimental results (including those not listed) are considerable. In particular, the differences between the four different theoretical calculations are even more obvious [4,8,21]. Compared with other theoretical calculation results, the results of this study are in better agreement with the experimental values, especially when the temperature exceeds 100 °C. There are a lot of experimental studies on oxygen isotope fractionation among carbonate minerals, such as the works of Northrop and Clayton, Clayton et al., and the relevant data reported in Chacko and Deines’s work, etc. [8,35,36]. Those who are interested can refer to the relevant data. Sheppard and Schwarcz investigated the fractionation of oxygen isotopes in coexisting metamorphic calcite and dolomite by measuring natural samples [37]. Their experimental results show that the oxygen isotope fractionation factor between dolomite and calcite has a temperature function relationship of $1000\ln\alpha_{Dolomite-Calcite}^{18_{O}}=0.45x-0.40$ ($x=10^{6}/T^{2}$) in the temperature range of 100–600 °C. Matthews and Katz experimentally studied the oxygen isotope fractionation during the dolomitization of calcium carbonate at 252, 265, 274, 285, and 295 °C [32]. Although their experimental data were limited (only five data points), they were able to illustrate the oxygen isotope fractionation characteristics of the two carbonate minerals at temperatures ranging from 252 to 295 °C (see Figure 7). Our theoretical calculation results are slightly greater than their experimental results and better than other theoretical results reported by predecessors. This difference may result from their choice of experimental method, especially the choice of experimental solution concentrations and the Mg/Ca ratio. In the experiment of Matthews and Katz, an Mg/Ca ratio of 0.26 was selected, and increasing or decreasing Mg/Ca ratios may affect the final determination of oxygen isotope fractionation between carbonate minerals [32].

Zheng systematically calculated the oxygen isotope fractionation factors of carbonate minerals and sulfate minerals by an increment method [4]. As can be seen from Figure 7, the research results of Zheng are significantly different from the results of this study, Schauble et al. and Chacko and Deines [4,8,21]. The increment method has been proved by many studies to be unsuitable for the study of various solid mineral systems [8,22]. Chacko and Deines’s work give a range that is determined by different Mg/(Mg + Ca) ratios. Those who are interested can see Figure 7 in this article for more details [8].

#### 3.4.2. Oxygen Isotope Fractionation Factors between Carbonate Minerals and Aqueous Solutions

**Figure 8:**
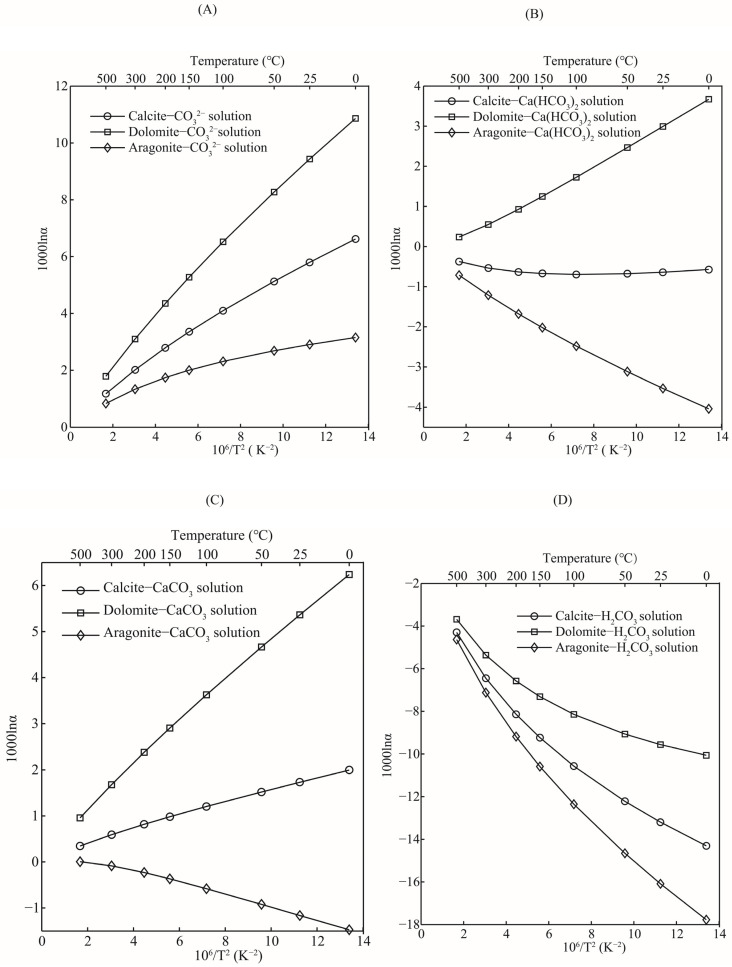
Relations of oxygen isotope fractionation factors between minerals (calcite, dolomite, and aragonite) and aqueous solutions (H_2_CO_3_ solution, CO_3_^2−^ solution, CaCO_3_ solution, and Ca(HCO_3_)_2_ solution) with temperature. (**A**–**D**) stand for the function diagrams between oxygen isotope fractionation factors and temperature for pairs of carbonate minerals-CO_3_^2−^ solution, carbonate minerals-Ca(HCO_3_)_2_ solution, carbonate minerals-CaCO_3_ solution and carbonate minerals-H_2_CO_3_ solution, respectively.

The oxygen isotope fractionation factors between carbonate minerals and different aqueous solutions were systematically studied. Taking an H_2_CO_3_ aqueous solution as an example, in the theoretical calculation, its gas phase molecular form is H_2_CO_3_(g), while the aqueous solution species is H_2_CO_3_·nH_2_O (in this study H_2_CO_3_·30H_2_O was used). The purpose of this operation is to investigate the contribution of these different species to the oxygen isotope effects of carbonate dissolution and precipitation on a molecular basis. For the sake of discussion, oxygen isotope fractionation factors between three carbonate minerals (calcite, aragonite, and dolomite) and aqueous solutions are used as research objects. The oxygen isotope fractionation effect between other carbonate minerals and aqueous solutions can also be obtained through the same treatment process. As shown in Figure 8, the abilities of aqueous solutions to enrich heavy oxygen isotope (^18^O), with respect to the three carbonate minerals, were different. The overall change trend of oxygen isotope fractionation factors for these three systems was dolomite-aqueous solutions > calcite-aqueous solutions > aragonite-aqueous solutions (Figure 8A). This change order is consistent with the RPFRs of dolomite, calcite, and aragonite. In terms of oxygen isotope fractionation factors between carbonate minerals and CO_3_^2−^ solutions, these three minerals were more enriched with ^18^O than the CO_3_^2−^ solution. Among them, dolomite had the strongest ability to enrich heavy oxygen isotopes (^18^O). Taking the equilibrium oxygen isotope fractionation factors at 25 °C as an example, the 1000lnα_s_ for systems of dolomite-CO_3_^2−^ solution, calcite-CO_3_^2−^ solution, and aragonite-CO_3_^2−^ solution were 9.43, 5.79, and 2.90, respectively. The experimental results and published isotope fractionation data demonstrated that CO_3_^2−^ contributed significantly to the equilibrium oxygen isotope fractionation factors of carbonate minerals [38]. Devriendt et al.’s results show that $\alpha_{C-CO_{3}^{2-}}^{eq}$ (C stands for CaCO_3_) is 1.00542 at the temperature of 33.7 °C [11]. If you convert that to 1000lnα, it is equal to 5.42. This value is close to the oxygen isotope fractionation factor of a calcite-CO_3_^2−^ solution (5.79) but is quite different from the dolomite-CO_3_^2−^ solution (9.43) and aragonite-CO_3_^2−^ solution (2.90) systems. Therefore, it is essential to accurately determine the species of carbonate when calculating the oxygen isotope fractionation factor between carbonate and CO_3_^2−^ solutions. See Table 4 for the oxygen isotope fractionation factors at other temperatures.

Bicarbonate ions (HCO_3_^−^) also play an important role in the water–rock interaction between carbonate minerals and water. In the process of water–rock interactions, HCO_3_^−^ plays a crucial role as the controlling factor that determines the species balance of an aqueous solution [38]. The concentration of HCO_3_^−^ in aqueous solutions is controlled by multiple factors, such as pH, temperature, salinity, etc. [11]. When the pH value of the solution is low, or the concentration of carbon dioxide (CO_2_) in the surrounding atmosphere is high, the water–rock interaction process will move in the direction of the formation of HCO_3_^−^, that is, the dissolution of carbonate minerals will occur. Compared with the aqueous solution of HCO_3_^−^, the three carbonate minerals differed greatly in their ability to enrich heavy oxygen isotopes (^18^O). Dolomite was more enriched with ^18^O than the HCO_3_^−^ solution, while for calcite and aragonite, the HCO_3_^−^ solution was more enriched with ^18^O (Figure 8B). Similarly, the oxygen isotope fractionation factors of the dolomite-HCO_3_^−^ solution, calcite-HCO_3_^-^ solution, and aragonite-HCO_3_^−^ solution at 25 °C were 2.99, −0.63, and −3.53, respectively. Devriendt et al. conducted a systematic experimental study on the oxygen isotope fractionation of the CaCO_3_-DIC-H_2_O system [11]. Their work found that the oxygen isotope fractionation factor between carbonate-HCO_3_^−^ solutions is kinetically controlled. When considering equilibrium oxygen isotope fractionation, among the three carbonate minerals aragonite is the most depleted in ^18^O compared to HCO_3_^−^ solutions. The oxygen isotope fractionation factors of these three systems under other temperature conditions can also be obtained from Table 4.

Calcium carbonate (CaCO_3_) is in an aqueous solution, usually in the form of precipitation [29,39,40]. It is well known that calcium carbonate is insoluble in water, but it also has a solubility product constant Ksp, that is, the precipitation equilibrium constant, although this value is very small. When precipitation reaches the equilibrium state of precipitation-dissolution in solution, the concentration of each ion remains unchanged, and the product of the power of the ion concentration is a constant, which is called the solubility product constant. Although the solubility of calcium carbonate in aqueous solution is very low, the oxygen isotope fractionation factors between a calcium carbonate solution and minerals are systematically studied. The three carbonate minerals also differ in their ability to enrich ^18^O relative to CaCO_3_ solutions (Figure 8C). Dolomite and calcite are more enriched in heavy oxygen isotopes than CaCO_3_ solutions, while aragonite is depleted in heavy oxygen isotopes compared with CaCO_3_ solutions. At 25 °C, the oxygen isotope fractionation factors of dolomite-CaCO_3_ solution, calcite-CaCO_3_ solution, and aragonite-CaCO_3_ solution are 5.36, 1.72, and −1.16, respectively. Oxygen isotope fractionation factors at other temperatures are also given in detail in Table 4.

Carbonic acid (H_2_CO_3_) is a binary weak acid with the formula H_2_CO_3_ and has a relatively small ionization constant. Therefore, in the natural water body (seawater, surface water, or groundwater), there will also be an H_2_CO_3_ aqueous solution in the form of H_2_CO_3_ molecules. The existence of an H_2_CO_3_ solution has a crucial effect on the balance of the CO_2_ concentration [11] between water and the atmosphere, and the concentration of HCO_3_^−^ in an aqueous solution. Its existence, in fact, plays the role of a buffer. In turn, it will affect the water–rock interaction between the carbonate minerals and water. Compared with other aqueous solutions, an H_2_CO_3_ aqueous solution has a stronger ability to enrich heavy oxygen isotopes (Figure 8D). Dolomite, calcite, and aragonite are all depleted heavy oxygen isotopes relative to the H_2_CO_3_ aqueous solution. At 25 °C, the oxygen isotope fractionation factors of the dolomite-H_2_CO_3_ solution, calcite-H_2_CO_3_ solution, and aragonite-H_2_CO_3_ solution are −9.56, −13.19, and −16.09 respectively. In this chemical reaction process, the phenomenon of heavy oxygen isotope loss of solid minerals, relative to a carbonic acid aqueous solution, is very obvious. Even at higher temperatures, such as 500 °C, the oxygen isotope fractionation factors can still reach −3.68, −4.29, and −4.63. Please refer to Table 4 for oxygen isotope fractionation factors at other temperatures.

#### 3.4.3. Oxygen Isotope Fractionation between Aqueous Solutions and Gas Phases

**Figure 9:**
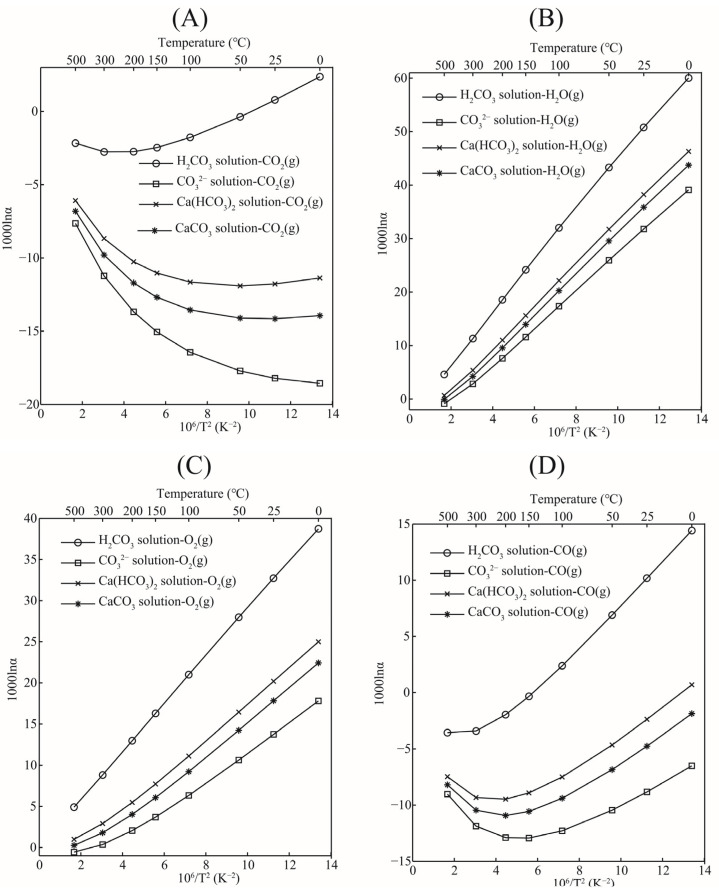
The graphs of oxygen isotope fractionation factors between aqueous solutions and gas phases as a function of temperature. (**A**): aqueous species-CO_2_(g); (**B**): aqueous species-H_2_O(g); (**C**): aqueous species-O_2_(g); (**D**): Aqueous species-CO(g).

The oxygen isotope fractionation factors between aqueous solutions (such as CO_3_^2−^ solutions, Ca(HCO_3_)_2_ solutions, CaCO_3_ solutions, and H_2_CO_3_ solutions) and oxygen-bearing gas phases (CO_2_(g), CO(g), O_2_(g), and H_2_O(g)) were systematically studied. The oxygen isotope fractionation factors between aqueous solutions and gas phases as a function of temperature are shown in Figure 9. The results showed that, in most cases, the CO_2_(g) had a stronger ability to enrich heavy oxygen isotoped (^18^O) than aqueous solutions. Only under low-temperature conditions (0–50 °C) should the H_2_CO_3_ solution be slightly enriched with heavy oxygen isotopes (^18^O) (Figure 9A). At about 50 °C, the enrichment sequence of heavy oxygen isotopes (^18^O) for the oxygen isotope fractionation factors between H_2_CO_3_ aqueous solutions and CO_2_ was reversed. At 25 °C, the oxygen isotope fractionation factors of the H_2_CO_3_ solution-CO_2_(g), CO_3_^2−^ solution-CO_2_(g), Ca(HCO_3_)_2_ solution-CO_2_(g), and aqueous CaCO_3_ solution-CO_2_(g) were 0.77, −18.21, −11.77 and −14.14, respectively. With the exception of the H_2_CO_3_ solution, the CO_3_^2−^ solution, Ca(HCO_3_)_2_ solution, and aqueous CaCO_3_ solution have a significant depletion of heavy oxygen isotopes (^18^O) relative to CO_2_(g). These depletions are still very obvious even at higher temperature conditions, such as 500 °C; this series of values can still reach −2.16, −7.63, −6.08, and −6.80 (‰) (See Table 5). Another conclusion can be drawn from Figure 9A, that is, the oxygen isotope fractionation factors between aqueous solution species and CO_2_(g) did not completely decrease gradually with the decrease in temperature. In general, except for the CO_3_^2−^ solution-CO_2_(g) system, oxygen isotope fractionation factors decreased first and then increased with the increase in temperature. It is just that the temperature conditions that changed were different for different systems. Oxygen isotope fractionation factors between aqueous solutions and CO_2_(g) at other temperatures can be obtained from Table 5.

For oxygen isotope fractionation factors between aqueous solutions and H_2_O(g), the content of H_2_O(g) in the atmosphere is relatively small, but most of the H_2_O(g) exists in the troposphere, and the relationship between the surface layers is very close. Almost all weather events in the troposphere are related to H_2_O(g). The oxygen isotope fractionation between the carbonic acid aqueous solution systems and H_2_O(g) is shown in Figure 9B. The H_2_CO_3_ solution, Ca(HCO_3_)_2_ solution, CaCO_3_ solution, and CO_3_^2−^ solution were all more enriched with heavy oxygen isotopes (^18^O) than H_2_O(g). Among them, the H_2_CO_3_ solution had the strongest ability to enrich heavy oxygen isotopes relative to H_2_O(g). At 25 °C, the oxygen isotope fractionation factors between the H_2_CO_3_ solution, Ca(HCO_3_)_2_ solution, CaCO_3_ solution, CO_3_^2−^ solution, and H_2_O(g) were 50.77, 38.22, 35.85, and 31.78, respectively. Data for oxygen isotope fractionation at other temperatures are shown in Table 5. The oxygen isotope fractionation factors of all systems decreased linearly with the increase in temperature.

Carbon monoxide and oxygen are also important oxygen carriers in the atmosphere. Oxygen isotope fractionation between aqueous solutions and these two gases has also been studied in detail. The relationship between the fractionation factors and temperature is shown in Figure 9C,D. The oxygen isotope fractionation factor between aqueous solutions and O_2_(g) decreased gradually with the increase in temperature. The isotope fractionation factor between H_2_CO_3_ solution and oxygen was the largest one. At 25 °C, The oxygen isotope fractionation factors of the H_2_CO_3_ solution-O_2_(g), CO_3_^2−^ solution-O_2_(g), Ca(HCO_3_)_2_ solution-O_2_(g), and CaCO_3_ solution-O_2_(g) were 32.74, 13.75, 20.18 and 17.81, respectively. The uniform pattern of change was that aqueous solutions were more enriched with heavy isotopes than O_2_(g). The oxygen isotope fractionation between the solutions and CO_2_(g) did not show a consistent decrease with the increase in temperature but would reverse when the temperature rises to a certain value. Compared with CO_2_(g), except for the H_2_CO_3_ solution which is more enriched with heavy isotope, the other solutions showed different degrees of heavy oxygen isotope depletion.

#### 3.4.4. Oxygen Isotope Fractionation between Aqueous Solutions

**Figure 10:**
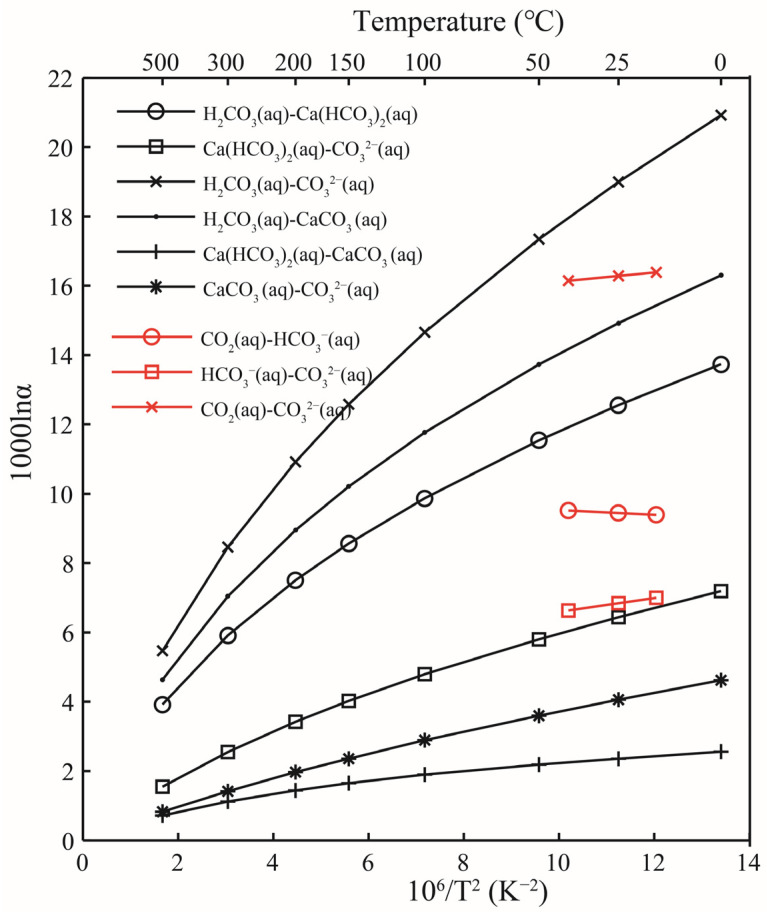
Relationship of oxygen isotope fractionation factors between different aqueous solutions as a function of temperature. The black and red lines represent the results of this study and Beck [7], respectively. “aq” stands for solution.

In the aqueous carbonate system, the isotope exchange reactions are shown in Formula (3). When constructing the molecular clusters and calculating the harmonic vibration frequencies, only one oxygen atom exchange reaction is considered. The oxygen isotope exchange reaction between the Ca(HCO_3_)_2_ solution and the CO_3_^2−^ solution occurs with the exchange of only one oxygen atom, as shown in the Formula (3). There have been many previous experimental studies on carbonic acid systems, such as Beck’s work [7]. In the work of Beck, oxygen isotope fractionation in the carbonic acid system at 15, 25, and 40 °C was systematically studied [7]. In the work of McCrea, the temperature variation of oxygen fractionation in the exchange reactions between dissolved carbonate and water and between calcite and water was investigated theoretically and experimentally [19]. At 25 °C, the oxygen isotope fractionation factor between the Ca(HCO_3_)_2_ solution and the CO_3_^2−^ solution was 6.43 (Table 6), and the results obtained by recalculating Beck et al. and McCrea were 6.81 and 6.28 [7,19], respectively. The data obtained in this study are in good agreement with the previous studies. For the oxygen isotope fractionation factors between the H_2_CO_3_ solution and the CO_3_^2−^ solution, and between the H_2_CO_3_ solution and the Ca(HCO_3_)_2_ solution, the results of this study were 18.99 and 12.55, respectively. The work by Beck et al. showed corresponding figures of 16.30 and 9.49, respectively. Our calculations were larger than the data from Beck et al. [7]. The reason for this gap may be that we use the structure H_2_CO_3_ when calculating the carbonic H_2_CO_3_ solution, while in the laboratory, they use CO_2_ (aq). CO_2_ is easily soluble in water, and the real existence structure should be in the form of H_2_CO_3_ and CO_2_ molecules. This work showed that the H_2_CO_3_ solution had the highest ability to enrich ^18^O among all solutions (Figure 10).

(3) $$\begin{array}{l} {\mathrm{HC}}^{16}O^{16}O_{2}^{-}+C^{18}O^{16}O_{2}^{2-}⇌{HC}^{18}O^{16}O_{2}^{-}+C^{16}O^{16}O_{2}^{2-}\\ H_{2}C^{16}O^{16}O_{2}+{HC}^{18}O^{16}O_{2}^{-}⇌H_{2}C^{18}O^{16}O_{2}+{HC}^{16}O^{16}O_{2}^{-}\\ H_{2}C^{16}O^{16}O_{2}+C^{18}O^{16}O_{2}^{2-}⇌H_{2}C^{18}O^{16}O_{2}+C^{16}O^{16}O_{2}^{2-} \end{array}$$

### 3.5. Applications of O Isotope Fractionation Factor

The oxygen isotope equilibrium fractionation factor between calcite and water is crucial for the accurate determination of the formation temperature of carbonate deposition, and there has been a lot of research on its application to ancient climate studies [18,19,41,42]. It is very important to obtain an accurate oxygen isotope fractionation factor for obtaining an accurate geological temperature. However, there are few studies on whether the oxygen isotope fractionation factors of carbonate minerals have other factors. It has been proved that the oxygen isotope fractionation factor of carbonate minerals is a function of HCO_3_^−^ and CO_3_^2−^ [7,43]. In this study, the oxygen isotope fractionation factors between aqueous solution species (other than water) and carbonate minerals were studied theoretically. In addition, the oxygen isotope exchange reaction between different species in aqueous solution and the oxygen isotope exchange reaction between aqueous solutions and gas phase species also play a crucial role in the water–rock interaction process of carbonate. At 25 °C, the results of this study showed that the difference between 1000ln_calcite-aqueous solutions_ and 1000ln_calcite-water_ was very significant. Previous experimental results show that the widely used value of 1000ln_calcite-water_ is 29.80 or 28.3 [34]. At the same temperature, the oxygen isotope fractionation factors between calcite and other aqueous species were −13.19 (calcite-H_2_CO_3_ solution), 5.79 (calcite-CO_3_^2−^ solution), −0.63 (calcite-Ca(HCO_3_)_2_ solution), and 1.72 (calcite-CaCO_3_ solution), respectively. Therefore, other aqueous species in an aqueous solution can significantly affect the oxygen isotopic composition of carbonate. In addition to considering the effect of water on the oxygen isotopic composition of carbonate minerals, other species in aqueous solutions such as CO_3_^2−^ and HCO_3_^−^ also must be considered. This is because CO_3_^2−^ and HCO_3_^−^ are the main species that are very important in aqueous solutions in carbonate regions (karst regions). Many processes in nature are mostly open processes, and the water–rock interaction is no exception. As one of the most critical factors, CO_2_(g) also profoundly affects the oxygen isotope exchange in this process. The results showed that the oxygen isotope fractionation factors between aqueous species and CO_2_(g) were 0.77 (H_2_CO_3_ solution-CO_2_(g)), −18.24 (CO_3_^2−^ solution-CO_2_(g)), −11.77 (Ca(HCO_3_)_2_ solution-CO_2_(g)), and −14.14 (CaCO_3_ solution-CO_2_(g)), respectively, at 25 °C. In other words, compared with aqueous species CO_3_^2−^ and HCO_3_^−^, CO_2_(g) was significantly enriched in heavy oxygen isotopes (^18^O). This process can significantly affect the oxygen isotopic composition of aqueous solution. The effects of different microscopic processes on oxygen isotope fractionation factors were systematically studied at the molecular level. These oxygen isotope parameters obtained in this study will provide the necessary theoretical support for explaining the oxygen isotope fractionation effect of the mineral-solution-gas phase.

In geological environments, the oxygen isotope exchange reaction between carbonate minerals and aqueous solutions may not achieve isotope exchange equilibrium. This could also be the cause of the differences between the isotopic fractionation factors calculated on theoretical grounds, determined via laboratory experiments, or measured in natural systems. In this study, the oxygen isotope equilibrium fractionation factors of different oxygen isotope exchange reactions were calculated based on the theoretical calculation. Therefore, there are always differences between the theoretical and experimental values, and these differences can confirm whether the oxygen isotope exchange reactions reach equilibrium in different geological processes.

## 4. Methodology

The equilibrium isotope fractionation factor is a mass-dependent parameter. The harmonic vibration frequencies of different isotopologues will change after the isotope substitution reaction for substance A, which is also the basis for calculating the equilibrium isotope fractionation factor [17,18]. This method has been elaborated in many research papers and reviews [30,31,44,45]. Meanwhile, the equilibrium isotope fractionation factor is a function of temperature. For the following isotope exchange reactions:

(4) $$A+B^{*}=A^{*}+B$$

The equilibrium isotope fractionation factor (α_A-B_) of this isotope exchange reaction can be obtained by the reduced partition function ratio (RPFR, or β factor) of substance A and substance B. According to previous studies [17,18], the RPFR is defined as follows:

(5) $$\mathrm{RPFR}=\beta =\left(\frac{s*}{s}\right)\prod_{i}^{3n-6}\frac{u_{i}}{u_{i}*}\frac{\exp\left(-u_{i}/2\right)}{\exp\left(-{u_{i}}^{*}/2\right)}\frac{1-\exp\left(-u_{i}*\right)}{1-\exp\left(-u_{i}\right)}$$

In the above formula, “s” represents the symmetric number of molecules or molecular cluster, “*” represents the molecule or molecular cluster containing heavy isotope atoms, “n” represents the number of atoms in the molecule or molecular cluster, and parameter “u_i_” is a function of the harmonic vibration frequencies, i.e., $u_{i}=\frac{h\nu_{i}}{k_{b}T}$. “v_i_” represents the harmonic vibration vibrational frequencies of substance, “h” stands for the Planck constant, K_b_ is the Boltzmann constant, and T is the Kelvin temperature. To more intuitively see the isotope changes between different substances, the parameter $1000ln\alpha_{A-B}$ is used to indicate the isotope fractionation factor between different substances because the isotope fractionation factors are usually small.

According to the above introduction, the key to calculating the equilibrium fractionation factor of stable isotopes is to accurately obtain the harmonic vibration frequencies of molecules (molecular clusters). However, obtaining the harmonic frequency of the condensed phase (solid and liquid phase) is a difficult task. In the volume variable molecular cluster method (VVCM), the mineral is trimmed to the required size and considered a large molecular cluster when calculating the harmonic vibration frequencies. When modeling with this method, there is a well-known N-N-N principle, that is, there should be at least two atom layers outside the atom of interest [46]. Because the isotope effect is a local effect, the greatest effect on the isotope is the atom closest to it. After the model is constructed by VVCM, virtual charges are added to the outermost atoms to hold them in place, while the interior of the entire mineral fragment is freely optimized. The distances between the outermost atom and the virtual charges can be freely adjusted. By adjusting this distance many times until the most stable structure (the lowest energy structure) is obtained, then the structure was used to calculate the RPFR. This is also why this calculation method is time-consuming.

When calculating the harmonic vibration frequencies of an aqueous solution, the “water drop method” is used to deal with the solvation effect of the aqueous solution. Taking the calculation of the harmonic vibration frequencies for CO_3_^2−^ aqueous solution as an example, it is necessary to simulate a series of CO_3_^2−^ molecular clusters with different amounts of water molecules. The way to execute this process is by taking 6H_2_O as a group and adding it around CO_3_^2−^. Then, the cluster structure is optimized with the Gauss software package (Revision B. 01) [47]. After the optimized structure is obtained, water molecules are continued to be added around it (6H_2_O as a group), that is, CO_3_^2−^·12H_2_O, CO_3_^2−^·18H_2_O, CO_3_^2−^·24H_2_O, and CO_3_^2−^·30H_2_O are obtained successively. The structure of CO_3_^2−^·30H_2_O was selected as the final structure for calculating the oxygen isotope fractionation factor. To obtain more accurate calculation results, each structure (such as CO_3_^2−^·30H_2_O) is calculated four times in parallel, and their arithmetic mean value is used as the final calculation result. Therefore, this calculation process is cumbersome and time-consuming. It is worth noting that in all structural optimization and harmonic vibration frequency calculations, a unified theoretical calculation basis is necessary. Otherwise, unreasonable theoretical calculation results will be produced. In this study, no scaling factor was used to calculate the RPFR of oxygen isotopes [48,49]. This is because the correction for the harmonic frequencies can be canceled out when calculating the RPFR [16].

## 5. Conclusions

In this paper, the equilibrium oxygen isotope fractionation factors of systems Carbonate-Solution-Gas were systematically studied. In the process of real carbonate deposition or growth, it is very rare for oxygen isotope exchange to reach equilibrium, and in most cases, kinetic effects play a key role. These key equilibrium oxygen isotope fractionation parameters obtained in this study will provide oxygen isotope constraints for the complex water–rock interaction process. Different carbonate minerals have different abilities to enrich heavy oxygen isotopes, and the ^18^O enrichment order was siderite > magnesite > smithsonite > ankerite ≈ dolomite > otavite > calcite > aragonite > strontianite. For the H_2_CO_3_ solution, HCO_3_^−^ solution, CO_3_^2−^ solution, and CaCO_3_ solution, the ^18^O enrichment ability was the H_2_CO_3_ solution > HCO_3_^−^ solution > CaCO_3_ solution > CO_3_^2−^ solution. When the temperature range was 0–500 °C, the oxygen isotope fractionation factors of the calcite-H_2_CO_3_ solution, calcite-CO_3_^2−^ solution, calcite-Ca(HCO_3_)_2_ solution, and calcite-CaCO_3_ solution ranged from −14.3 to −4.29, 6.61 to 1.17, −0.69 to −0.37, and 1.99 to 0.34, respectively. At the same temperature range, the oxygen isotope fractionation factors of the dolomite-H_2_CO_3_ solution, dolomite-CO_3_^2−^ solution, dolomite-Ca(HCO_3_)_2_ solution, dolomite-CaCO_3_ solution, aragonite-H_2_CO_3_ solution, aragonite-CO_3_^2−^ solution, aragonite-Ca(HCO_3_)_2_ solution, and aragonite-CaCO_3_ solution varied from −10.06 to −3.68, 10.86 to 1.78, 3.67 to 0.23, 6.23 to 0.95, −17.77 to −4.63, 3.15 to 0.83, −4.04 to −0.71, and −1.47 to 0.00.

## Figures and Tables

**Table 1:** Comparison of optimized carbonate mineral structure data obtained by theoretical calculation in this study with previous data.

Minerals	Bond Length (Å)							
	This Study	Previous Data	This Study	Previous Data	This Study	Previous Data	This Study	Previous Data
Calcite	O-C		O-Ca_1_		O-Ca_2_			
	1.28	1.28 ^a^	2.36	2.36 ^a^	2.37	2.36 ^a^		
Dolomite	O-C		O-Mg		O-Ca			
	1.28	1.23 ^a^	2.08	2.11 ^a^	2.40	2.40 ^a^		
Aragonite	C-O		O-Ca_1_		O-Ca_2_		O-Ca_3_	
	1.28	1.28 ^b^	2.62	2.65 ^b^	2.43	2.42 ^b^	2.77	2.65 ^b^
Ankerite	O-C		O-Ca		O-Fe			
	1.29	1.28 ^c^	2.40	2.37 ^c^	2.06	2.11 ^c^		
Magnesite	O-C		O-Mg_1_		O-Mg_2_			
	1.28	1.28 ^a^	2.13	2.10 ^a^	2.11	2.10 ^a^		
Nitratine	O-N		O-Na_1_		O-Na_2_			
	1.25	1.25 ^d^	2.40	2.40 ^d^	2.41	2.41 ^d^		
Otavite	C_O		O_Cd_1_		O_Cd_2_			
	1.28	1.28 ^a^	2.33	2.28 ^a^	2.31	2.28 ^a^		
Siderite	C-O		O-Fe_1_		O-Fe_2_			
	1.29	1.29 ^a^	2.10	2.10 ^a^	2.08	2.08 ^a^		
Smithsonite	C-O		O-Zn_1_		O-Zn_2_			
	1.28	1.28 ^a^	2.14	2.11 ^a^	2.14	2.11 ^a^		
Strontianite	C-O		O-Sr_1_		O-Sr_2_		O-Sr_3_	
	1.29	1.29 ^b^	2.66	2.63 ^b^	2.69	2.66 ^b^	2.63	2.56 ^b^

Note: O-Ca_1_ and O-Ca_2_ represent different chemical bonds, as do the others. ^a^, ^b^, ^c^, and ^d^ represent the results of Graf, De Villiers, Reeder and Dollase, and Paul and Pryor [23,24,25,26], respectively.

**Table 2:** Reduced partition function ratios of different carbonate minerals, aqueous solutions, and gas phase species at different temperatures.

	Temperature (°C)	0	25	50	100	150	200	300	500
Species									
Calcite		116.42	101.48	89.21	70.43	56.93	46.90	33.32	19.15
Aragonite		112.95	98.59	86.77	68.65	55.58	45.85	32.64	18.81
Dolomite		120.66	105.11	92.36	72.85	58.85	48.46	34.40	19.77
Ankerite		120.97	105.30	92.45	72.83	58.76	48.34	34.27	19.65
Magnesite		125.64	109.41	96.10	75.78	61.19	50.39	35.76	20.55
Otavite		117.68	102.50	90.05	71.01	57.35	47.21	33.50	19.23
Siderite		126.33	109.81	96.29	75.70	60.98	50.11	35.46	20.29
Smithsonite		122.76	106.88	93.86	73.98	59.72	49.16	34.87	20.02
Strontianite		109.74	95.70	84.15	66.47	53.74	44.28	31.46	18.09
H_2_CO_3_ solution		130.73	114.68	101.43	81.00	66.16	55.04	39.77	23.45
CO_3_^2−^ solution		109.80	95.68	84.08	66.33	53.58	44.11	31.31	17.98
Ca(HCO_3_)_2_ solution		116.99	102.12	89.89	71.13	57.60	47.53	33.86	19.53
CaCO_3_ solutions		114.42	99.75	87.69	69.23	55.94	46.08	32.73	18.81
CO_2_		128.36	113.90	101.80	82.78	68.63	57.79	42.53	25.62
CO		116.31	104.51	94.54	78.63	66.51	57.02	43.18	27.01
O_2_		92.00	81.93	73.45	60.00	49.88	42.06	30.94	18.54

**Table 3:** The formula for calculating the ratio of the reduced partition function for oxygen isotopes (^18^O/^16^O) of different carbonate minerals, aqueous solutions, and gas phase species ($1000\ln RPFR=Ax^{3}+Bx^{2}+Cx+D$, *A*, *B*, *C*, and *D* are the corresponding parameter terms respectively).

	Temperature (°C)	*A*	*B*	*C*	*D*
Species 1000ln*RPFR*					
Calcite		0.007230263	−0.327071082	11.75192151	0.300504393
Aragonite		0.007537059	−0.334833352	11.54025831	0.327382137
Dolomite		0.007330701	−0.332446906	12.12218913	0.305139189
Ankerite		0.006954465	−0.320666722	12.05684711	0.276942952
Magnesite		0.007600885	−0.343589381	12.593581	0.322006359
Otavite		0.007031426	−0.320985282	11.80127156	0.285032674
Siderite		0.006813084	−0.31770406	12.44337763	0.267189992
Smithsonite		0.007290082	−0.331628155	12.27563624	0.301457795
Strontianite		0.006841885	−0.310558345	11.10316461	0.282024677
H_2_CO_3_ solution		0.012347405	−0.491138303	14.04917458	1.027100881
CO_3_^2−^ solution		0.00652037	−0.300327221	11.02900773	0.270182608
Ca(HCO_3_)_2_ solution		0.007899337	−0.349098715	11.9655552	0.360591326
CaCO_3_ solution		0.006983295	−0.319010134	11.53990994	0.290828537
CO_2_		0.018251757	−0.677288365	15.2748099	1.509108348
CO		0.025855553	−0.876549866	15.6063981	2.599607735
O_2_		0.013846506	−0.51859474	11.271756	0.851821871
H_2_O(g)		0.018608945	−0.599835355	9.724561979	3.531922144

**Table 4:** Oxygen isotope fractionation factors between carbonate minerals and aqueous solutions at different temperatures.

	Temperature (°C)	0	25	50	100	150	200	300	500
1000lnα (Mineral-Solution)									
Calcite-H_2_CO_3_ solution		−14.30	−13.19	−12.21	−10.56	−9.23	−8.13	−6.44	−4.29
Dolomite-H_2_CO_3_ solution		−10.06	−9.56	−9.06	−8.14	−7.31	−6.57	−5.36	−3.68
Aragonite-H_2_CO_3_ solution		−17.77	−16.09	−14.65	−12.35	−10.58	−9.18	−7.12	−4.63
Calcite-CO_3_^2−^ solution		6.61	5.79	5.12	4.09	3.34	2.78	2.01	1.17
Dolomite-CO_3_^2−^ solution		10.86	9.43	8.27	6.51	5.27	4.34	3.09	1.78
Aragonite-CO_3_^2−^ solution		3.15	2.90	2.68	2.31	2.00	1.74	1.33	0.83
Calcite-Ca(HCO_3_)_2_ solution		−0.57	−0.63	−0.67	−0.69	−0.67	−0.63	−0.53	−0.37
Dolomite-Ca(HCO_3_)_2_ solution		3.67	2.99	2.46	1.72	1.24	0.92	0.54	0.23
Aragonite-Ca(HCO_3_)_2_ solution		−4.04	−3.53	−3.11	−2.48	−2.02	−1.67	−1.21	−0.71
Calcite-CaCO_3_ solution		1.99	1.72	1.51	1.20	0.98	0.81	0.59	0.34
Dolomite-CaCO_3_ solution		6.23	5.36	4.66	3.62	2.90	2.37	1.67	0.95
Aragonite-CaCO_3_ solution		−1.47	−1.16	−0.92	−0.58	−0.36	−0.22	−0.08	0.00

**Table 5:** Oxygen isotope fractionation factors (in the form of 1000lnα) between aqueous solution species and gas phase species (CO_2_(g), CO(g), O_2_(g), and H_2_O(g)) at different temperatures.

	Temperature (°C)	0	25	50	100	150	200	300	500
Solution-Gas Pair									
H_2_CO_3_ solution-CO_2_(g)		2.36	0.77	−0.36	−1.77	−2.46	−2.75	−2.75	−2.16
CO_3_^2−^ solution-CO_2_(g)		−18.55	−18.21	−17.71	−16.44	−15.04	−13.67	−11.22	−7.63
Ca(HCO_3_)_2_ solution-CO_2_(g)		−11.36	−11.77	−11.90	−11.64	−11.02	−10.25	−8.67	−6.08
CaCO_3_ solution-CO_2_(g)		−13.93	−14.14	−14.10	−13.54	−12.68	−11.70	−9.79	−6.80
H_2_CO_3_ solution-CO(g)		14.41	10.16	6.88	2.37	−0.35	−1.97	−3.41	−3.55
CO_3_^2−^ solution-CO(g)		−6.50	−8.82	−10.45	−12.29	−12.93	−12.90	−11.87	−9.02
Ca(HCO_3_)_2_ solution-CO(g)		0.68	−2.38	−4.64	−7.49	−8.91	−9.48	−9.32	−7.47
CaCO_3_ solution-CO(g)		−1.88	−4.75	−6.84	−9.39	−10.56	−10.93	−10.45	−8.19
H_2_CO_3_ solution-O_2_(g)		38.72	32.74	27.97	20.99	16.28	12.98	8.82	4.90
CO_3_^2−^ solution-O_2_(g)		17.80	13.75	10.63	6.33	3.69	2.05	0.36	−0.56
Ca(HCO_3_)_2_ solution-O_2_(g)		24.99	20.18	16.43	11.12	7.72	5.47	2.91	0.98
CaCO_3_ solution-O_2_(g)		22.42	17.81	14.24	9.22	6.06	4.02	1.78	0.26
H_2_CO_3_ solution-H_2_O(g)		60.02	50.77	43.27	32.01	24.16	18.52	11.28	4.58
CO_3_^2−^ solution-H_2_O(g)		39.09	31.78	25.93	17.35	11.57	7.59	2.81	−0.88
Ca(HCO_3_)_2_ solution-H_2_O(g)		46.28	38.22	31.73	22.14	15.59	11.01	5.36	0.66
CaCO_3_ solution-H_2_O(g)		43.72	35.85	29.53	20.24	13.94	9.56	4.24	−0.05

**Table 6:** Oxygen isotope fractionation factors between different aqueous solutions at different temperatures.

	Temperature (°C)	0	25	50	100	150	200	300	500
Pairs									
H_2_CO_3_ solution-Ca(HCO_3_)_2_ solution		13.73	12.55	11.53	9.87	8.56	7.50	5.91	3.92
H_2_CO_3_ solution-CO_3_^2−^ solution		20.92	18.99	17.34	14.66	12.58	10.92	8.46	5.47
H_2_CO_3_ solution-CaCO_3_ solution		16.30	14.92	13.73	11.77	10.21	8.95	7.04	4.64
Ca(HCO_3_)_2_ solution-CO_3_^2−^ solution		7.19	6.43	5.80	4.79	4.02	3.42	2.54	1.55
Ca(HCO_3_)_2_ solution-CaCO_3_ solution		2.56	2.36	2.19	1.90	1.65	1.44	1.12	0.72
CaCO_3_ solution-CO_3_^2−^ solution		4.62	4.06	3.60	2.89	2.36	1.97	1.42	0.83

## Data Availability

Data are contained within the article.

## References

[1] O’Neil J.R. (1968). Hydrogen and oxygen isotope fractionation between ice and water. J. Phys. Chem..

[2] Brenninkmeijer C., Kraft P., Mook W. (1983). Oxygen isotope fractionation between CO_2_ and H_2_O. Chem. Geol..

[3] Swart P.K. (1983). Carbon and oxygen isotope fractionation in scleractinian corals: A review. Earth-Sci. Rev..

[4] Zheng Y.-F. (1999). Oxygen isotope fractionation in carbonate and sulfate minerals. Geochem. J..

[5] Chomicki K., Schiff S. (2008). Stable oxygen isotopic fractionation during photolytic O_2_ consumption in stream waters. Sci. Total Environ..

[6] Valley J.W., Bindeman I.N., Peck W.H. (2003). Empirical calibration of oxygen isotope fractionation in zircon. Geochim. Cosmochim. Acta.

[7] Beck W.C., Grossman E.L., Morse J.W. (2005). Experimental studies of oxygen isotope fractionation in the carbonic acid system at 15°, 25°, and 40 °C. Geochim. Cosmochim. Acta.

[8] Chacko T., Deines P. (2008). Theoretical calculation of oxygen isotope fractionation factors in carbonate systems. Geochim. Cosmochim. Acta.

[9] Thiemens M.H., Jackson T., Mauersberger K., Schueler B., Morton J. (2012). Oxygen isotope fractionation in stratospheric CO_2_. Geophys. Res. Lett..

[10] Zhang Y., Shi P., Song J., Li Q. (2018). Application of Nitrogen and Oxygen Isotopes for Source and Fate Identification of Nitrate Pollution in Surface Water: A Review. Appl. Sci..

[11] Devriendt L.S., Watkins J.M., McGregor H.V. (2017). Oxygen isotope fractionation in the CaCO_3_-DIC-H_2_O system. Geochim. Cosmochim. Acta.

[12] Guo W., Zhou C. (2019). Triple oxygen isotope fractionation in the DIC-H_2_O-CO_2_ system: A numerical framework and its implications. Geochim. Cosmochim. Acta.

[13] He H.-T., Liu Y. (2015). Silicon isotope fractionation during the precipitation of quartz and the adsorption of H4SiO4(aq) on Fe(III)-oxyhydroxide surfaces. Chin. J. Geochem..

[14] He H.-T., Zhang S., Zhu C., Liu Y. (2015). Equilibrium and kinetic Si isotope fractionation factors and their implications for Si isotope distributions in the Earth’s surface environments. Acta Geochim..

[15] Zhang J., Liu Y. (2018). Zinc isotope fractionation under vaporization processes and in aqueous solutions. Acta Geochim..

[16] Gao C., Cao X., Liu Q., Yang Y., Zhang S., He Y., Tang M., Liu Y. (2018). Theoretical calculation of equilibrium Mg isotope fractionations between minerals and aqueous solutions. Chem. Geol..

[17] Bigeleisen J., Mayer M.G. (1947). Calculation of Equilibrium Constants for Isotopic Exchange Reactions. J. Chem. Phys..

[18] Urey H.C. (1947). The thermodynamic properties of isotopic substances. J. Chem. Soc..

[19] McCrea J.M. (1950). On the Isotopic Chemistry of Carbonates and a Paleotemperature Scale. J. Chem. Phys..

[20] O’Neil J.R., Clayton R.N., Mayeda T.K. (1969). Oxygen Isotope Fractionation in Divalent Metal Carbonates. J. Chem. Phys..

[21] Schauble E.A., Ghosh P., Eiler J.M. (2006). Preferential formation of 13C–18O bonds in carbonate minerals, estimated using first-principles lattice dynamics. Geochim. Cosmochim. Acta.

[22] Schauble E.A., Young E.D. (2021). Mass Dependence of Equilibrium Oxygen Isotope Fractionation in Carbonate, Nitrate, Oxide, Perchlorate, Phosphate, Silicate, and Sulfate Minerals. Rev. Miner. Geochem..

[23] Graf D.L. (1961). Crystallographic tables for the rhombohedral carbonates. Am. Mineral..

[24] De Villiers J.P.R. (1971). Crystal Structures of Aragonite, Strontianite, and Witherite. Am. Mineral..

[25] Reeder R.J., Dollase W.A. (1989). Structural variation in the dolomite-ankerite solid-solution series; an X-ray, Moessbauer, and TEM study. Am. Mineral..

[26] Paul G.L., Pryor A.W. (1972). The study of sodium nitrate by neutron diffraction. Acta Crystallogr. Sect. B Struct. Crystallogr. Cryst. Chem..

[27] Demeny A., Kele S., Siklósy Z. (2010). Empirical equations for the temperature dependence of calcite-water oxygen isotope fractionation from 10 to 70 degrees C. Rapid Commun. Mass Spectrom..

[28] Zheng Y.-F. (2011). On the theoretical calculations of oxygen isotope fractionation factors for carbonate-water systems. Geochem. J..

[29] Lopez-Berganza J.A., Diao Y., Pamidighantam S., Espinosa-Marzal R.M. (2015). Ab Initio Studies of Calcium Carbonate Hydration. J. Phys. Chem. A.

[30] Richet P., Bottinga Y., Javoy M. (1977). A Review of Hydrogen, Carbon, Nitrogen, Oxygen, Sulphur, and Chlorine Stable Isotope Fractionation Among Gaseous Molecules. Annu. Rev. Earth Planet. Sci..

[31] Liu Q., Tossell J.A., Liu Y. (2010). On the proper use of the Bigeleisen–Mayer equation and corrections to it in the calculation of isotopic frac-tionation equilibrium constants. Geochim. Cosmochim. Acta.

[32] Matthews A., Katz A. (1977). Oxygen isotope fractionation during the dolomitization of calcium carbonate. Geochim. Cosmochim. Acta.

[33] Matthews A. (1994). Oxygen isotope geothermometers for metamorphic rocks. J. Metamorph. Geol..

[34] Coplen T.B. (2007). Calibration of the calcite–water oxygen-isotope geothermometer at Devils Hole, Nevada, a natural laboratory. Geochim. Cosmochim. Acta.

[35] Northrop D.A., Clayton R.N. (1966). Oxygen-Isotope Fractionations in Systems Containing Dolomite. J. Geol..

[36] Clayton R.N., Jones B.F. (1968). Isotope studies of dolomite formation under sedimentary conditions. Geochim. Cosmochim. Acta.

[37] Sheppard S.M.F., Schwarcz H.P. (1970). Fractionation of carbon and oxygen isotopes and magnesium between coexisting metamorphic calcite and dolomite. Contrib. Miner. Pet..

[38] Geisler T., Perdikouri C., Kasioptas A., Dietzel M. (2012). Real-time monitoring of the overall exchange of oxygen isotopes between aqueous CO32- and H_2_O by Raman spectroscopy. Geochim. Cosmochim. Acta.

[39] Wallace A.F., Hedges L.O., Fernandez-Martinez A., Raiteri P., Gale J.D., Waychunas G.A., Whitelam S., Banfield J.F., De Yoreo J.J. (2013). Microscopic Evidence for Liquid-Liquid Separation in Supersaturated CaCO_3_ Solutions. Science.

[40] Kilic S., Toprak G., Ozdemir E. (2016). Stability of CaCO_3_ in Ca(OH)_2_ solution. Int. J. Miner. Process..

[41] Epstein S., Buchsbaum R., Lowenstam H.A., Urey H.C. (1953). Revised Carbonate-water isotopic temperature scale. GSA Bull..

[42] Veizer J., Fritz P., Jones B. (1986). Geochemistry of brachiopods: Oxygen and carbon isotopic records of Paleozoic oceans. Geochim. Cosmochim. Acta.

[43] Kim S.-T., O’Neil J.R. (1997). Equilibrium and nonequilibrium oxygen isotope effects in synthetic carbonates. Geochim. Cosmochim. Acta.

[44] Schauble E.A. (2004). Applying Stable Isotope Fractionation Theory to New Systems. Rev. Miner. Geochem..

[45] Li X., Zhao H., Tang M., Liu Y. (2009). Theoretical prediction for several important equilibrium Ge isotope fractionation factors and geological im-plications. Earth Planet. Sci. Lett..

[46] Gibbs G.V. (1982). Molecules as models for bonding in silicates. Am. Mineral..

[47] Frisch M., Trucks G., Schlegel H., Scuseria G., Robb M., Cheeseman J., Scalmani G., Barone V., Petersson G., Nakatsuji H. (2009). Gaussian 16 Revision B. 01.

[48] Merrick J.P., Moran D., Radom L. (2007). An Evaluation of Harmonic Vibrational Frequency Scale Factors. J. Phys. Chem. A.

[49] Alecu I.M., Zheng J., Zhao Y., Truhlar D.G. (2010). Computational Thermochemistry: Scale Factor Databases and Scale Factors for Vibrational Frequencies Obtained from Electronic Model Chemistries. J. Chem. Theory Comput..

